# Supplementary material for: Structural basis for modulation of human NaV1.3 by clinical drug and selective antagonist
Source: Nat Commun. 2022 Mar 11;13:1286. doi: 10.1038/s41467-022-28808-5 (PMC8917200; doi:10.1038/s41467-022-28808-5)
Supplement: Supplementary file 1 — Supplementary Information [file 41467_2022_28808_MOESM1_ESM.pdf]

Supplementary Information for

Structural basis for modulation of human $Na_V1.3$ by clinical drug and selective antagonist

Xiaojing Li, Feng Xu, Hao Xu, Shuli Zhang, Yiwei Gao, Hongwei Zhang, Yanli Dong, Yanchun Zheng, Bei Yang, Jianyuan Sun, Xuejun Cai Zhang, Yan Zhao, Daohua Jiang

This file contains Supplementary Figure 1-8 and Table 1-3.

a

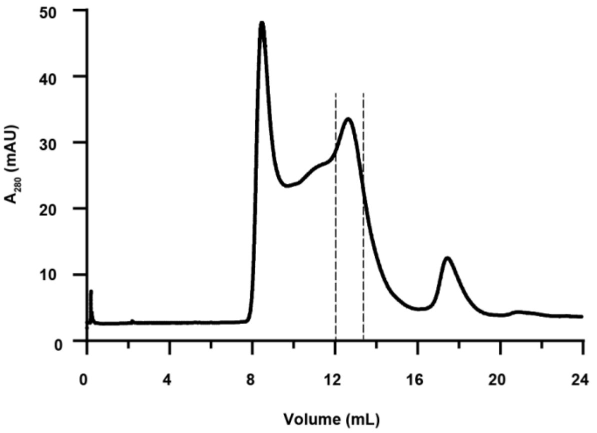

b

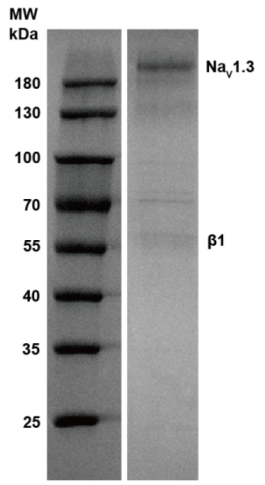

Supplementary Figure 1 Purification of the $Na_V1.3$-β1-β2 complex.

a. A representative size-exclusion chromatogram profile of the purified $Na_V1.3$-β1-β2 complex. Peak fractions labelled by black dashed lines were collected and concentrated for cryo-EM study. **b.** The purified sample of $Na_V1.3$-β1-β2 complex was stained by Coomassie blue on SDS-PAGE gel. $Na_V1.3$ and β1 are labelled. The band of β2 is smear due to glycosylation. The experiments were repeated independently with more than 3 times with similar results. Source data are provided as a Source Data file.

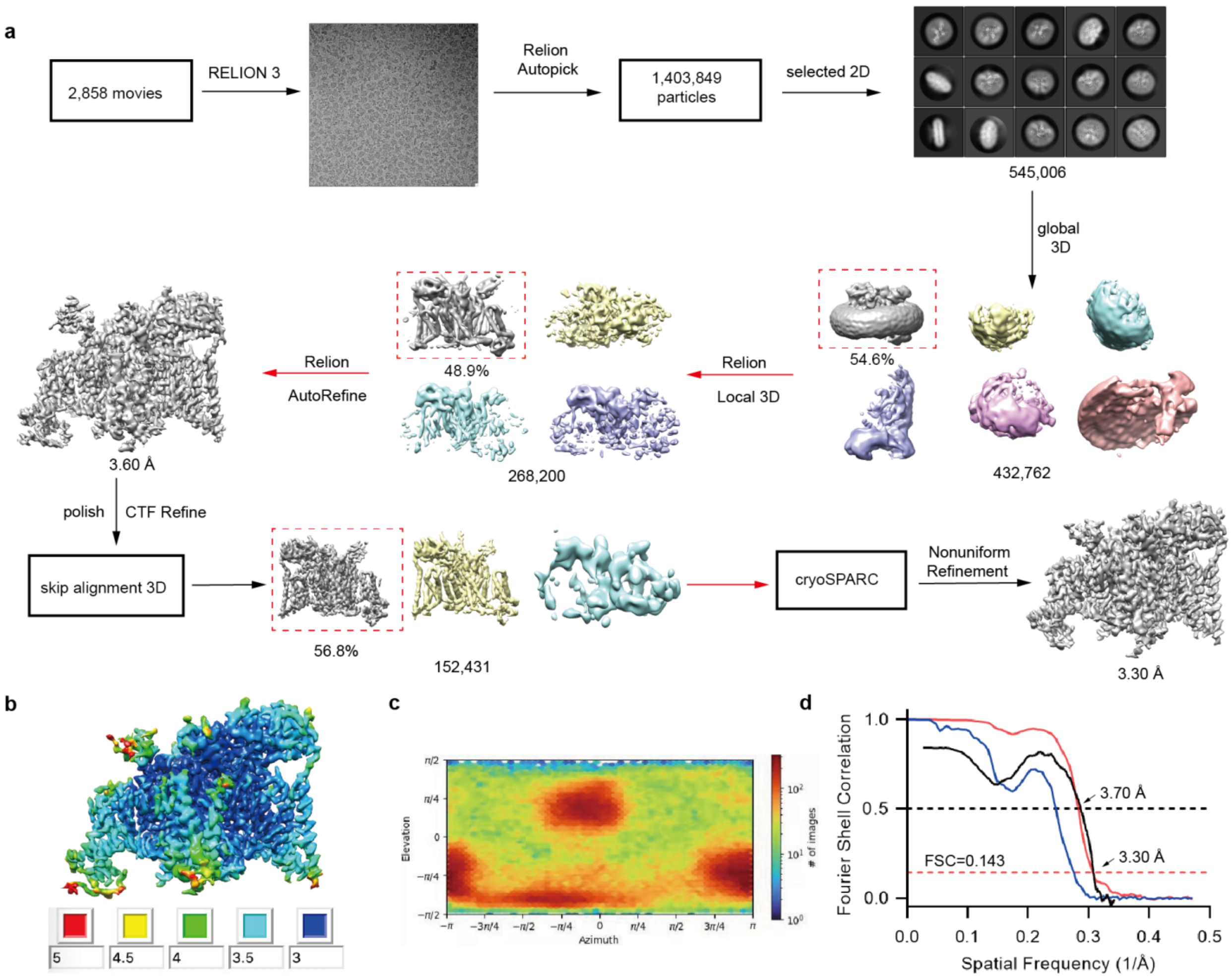

Supplementary Figure 2 Cryo-EM data processing of Na$_V$1.3-β1-β2-BLA complex.

a. Workflow of cryo-EM data processing. A total of 1,403,849 particles were selected from 2,858 movies. A representative motion-corrected micrograph of this dataset is shown here. Particles were picked using Relion, 2D and 3D classifications were conducted to remove bad particles, followed by Relion AutoRefine, Polish and CTF Refine to improve image quality. The final map was determined at 3.3 Å according to the gold-standard Fourier Shell Correlations (FSC) criterion. **b.** Sharpened map of the Na$_V$1.3 complex, colored according to the local resolution values. **c.** Particle angular distribution for the final 3D reconstruction. **d.** FSC of the final map of the Na$_V$1.3-β1-β2-BLA complex, calculated between two independently refined half-maps before (red) and after (blue) post-processing, overlaid with an FSC curve calculated between the cryo-EM density map and the structural model shown in black.

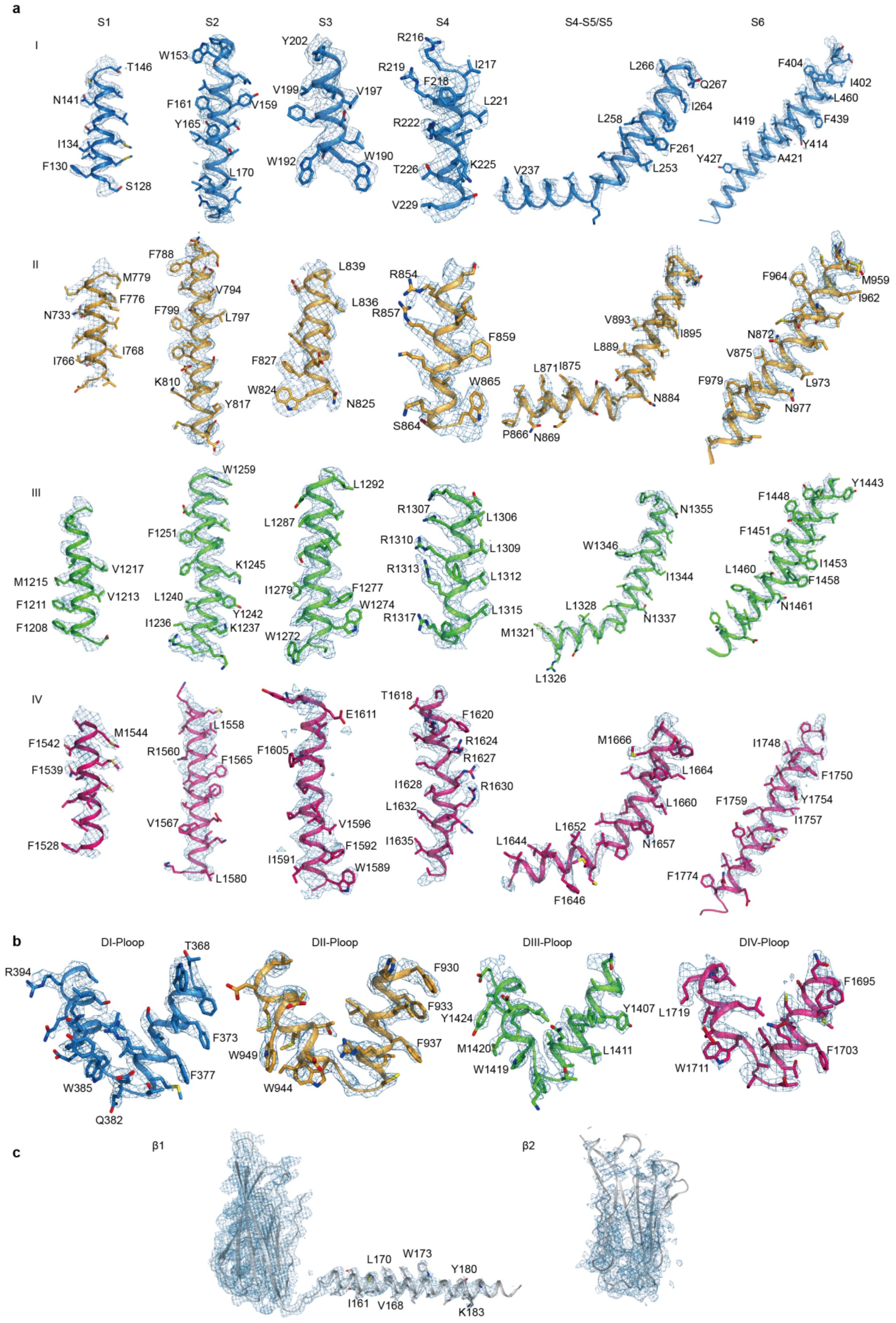

Supplementary Figure 3 EM maps for the Na$_V$1.3-β1-β2-BLA complex.

a. The EM density for each segment of each domain (S1-S6) in Na$_V$1.3 are shown in blue mesh, respectively. **b.** The P-loop of each domain is displayed. **C.** The auxiliary β1 and β2 subunits in the Na$_V$1.3 complex are shown individually. Side chain of residues with good density are shown in sticks. The same color code is applied as Figure 1b.

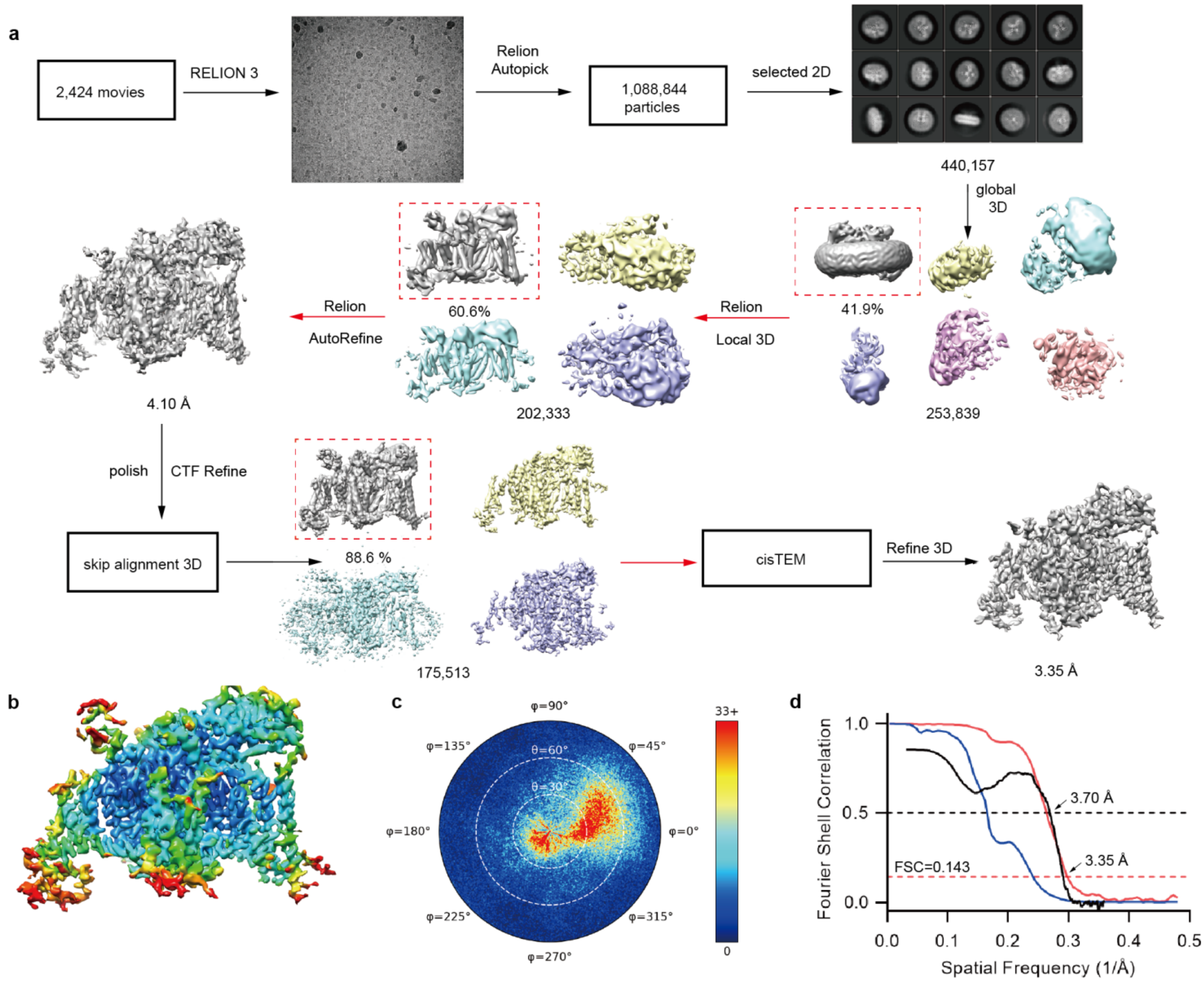

Supplementary Figure 4 Cryo-EM data processing of Na_V1.3-β1-β2-ICA complex.

a. Workflow of cryo-EM data processing. A total of 1,088,844 particles were selected from 2,424 movies. A representative motion-corrected micrograph of this dataset is shown here. Particles were picked using Relion, 2D and 3D classifications were conducted to remove bad particles, followed by Relion AutoRefine, Polish and CTF Refine to improve image quality. The final map was determined at 3.3 Å according to the gold-standard Fourier Shell Correlations (FSC) criterion. **b.** Sharpened map of the Na_V1.3 complex, colored according to the local resolution values. **c.** Particle angular distribution for the final 3D reconstruction. **d.** FSC of the final map of the Na_V1.3 complex, calculated between two independently refined half-maps before (red) and after (blue) post-processing, overlaid with an FSC curve calculated between the cryo-EM density map and the structural model shown in black.

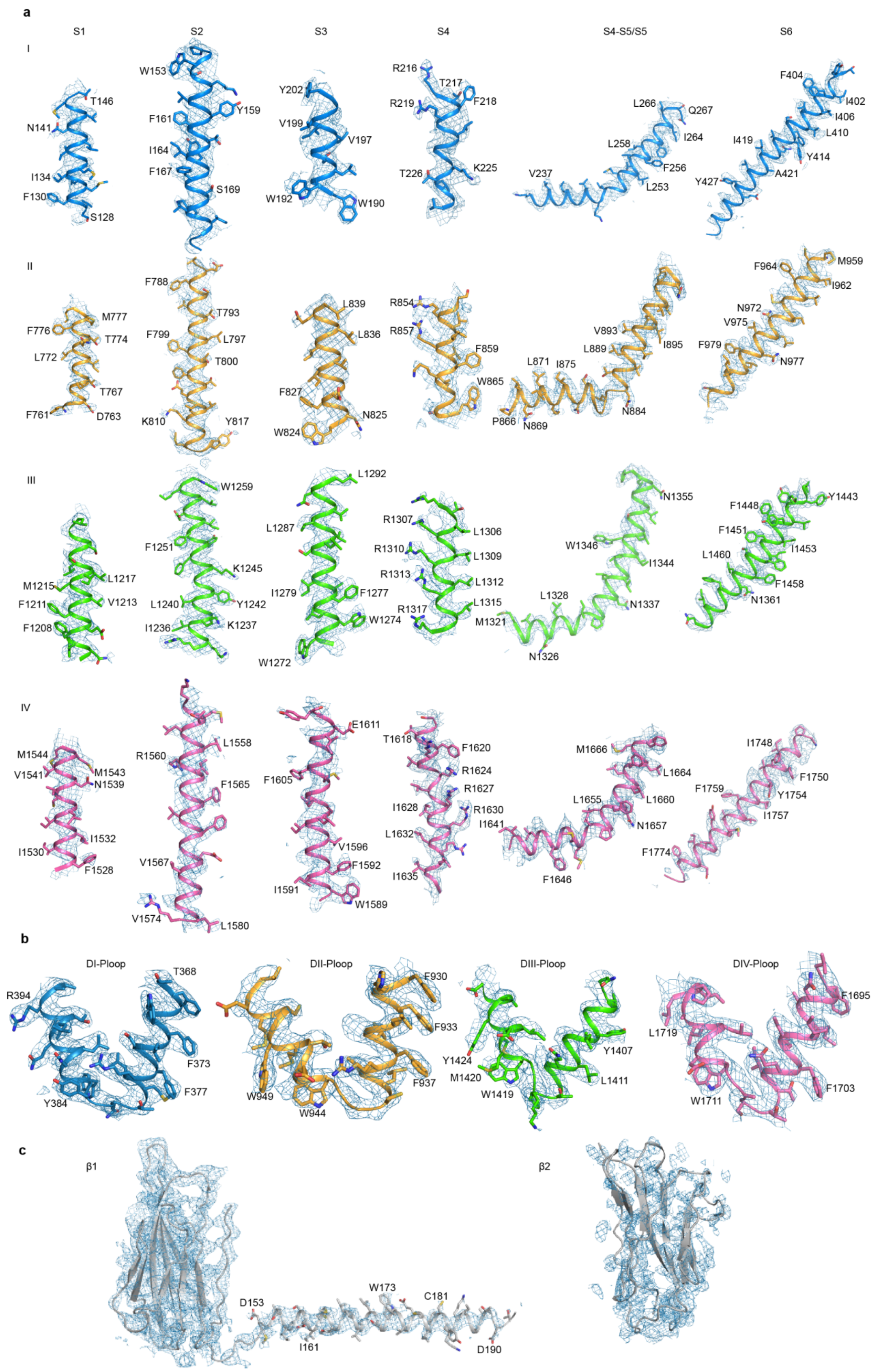

Supplementary Figure 5 EM maps for the $Na_V1.3$-β1-β2-ICA complex.

a. The EM density for each segment of each domain (S1-S6) in $Na_V1.3$ are shown in blue mesh, respectively. **b.** The P-loop of each domain is displayed. **C.** The auxiliary β1 and β2 subunits in the $Na_V1.3$ complex are shown individually. Side chain of residues with good density are shown in sticks. The same color code is applied as Figure 1b.

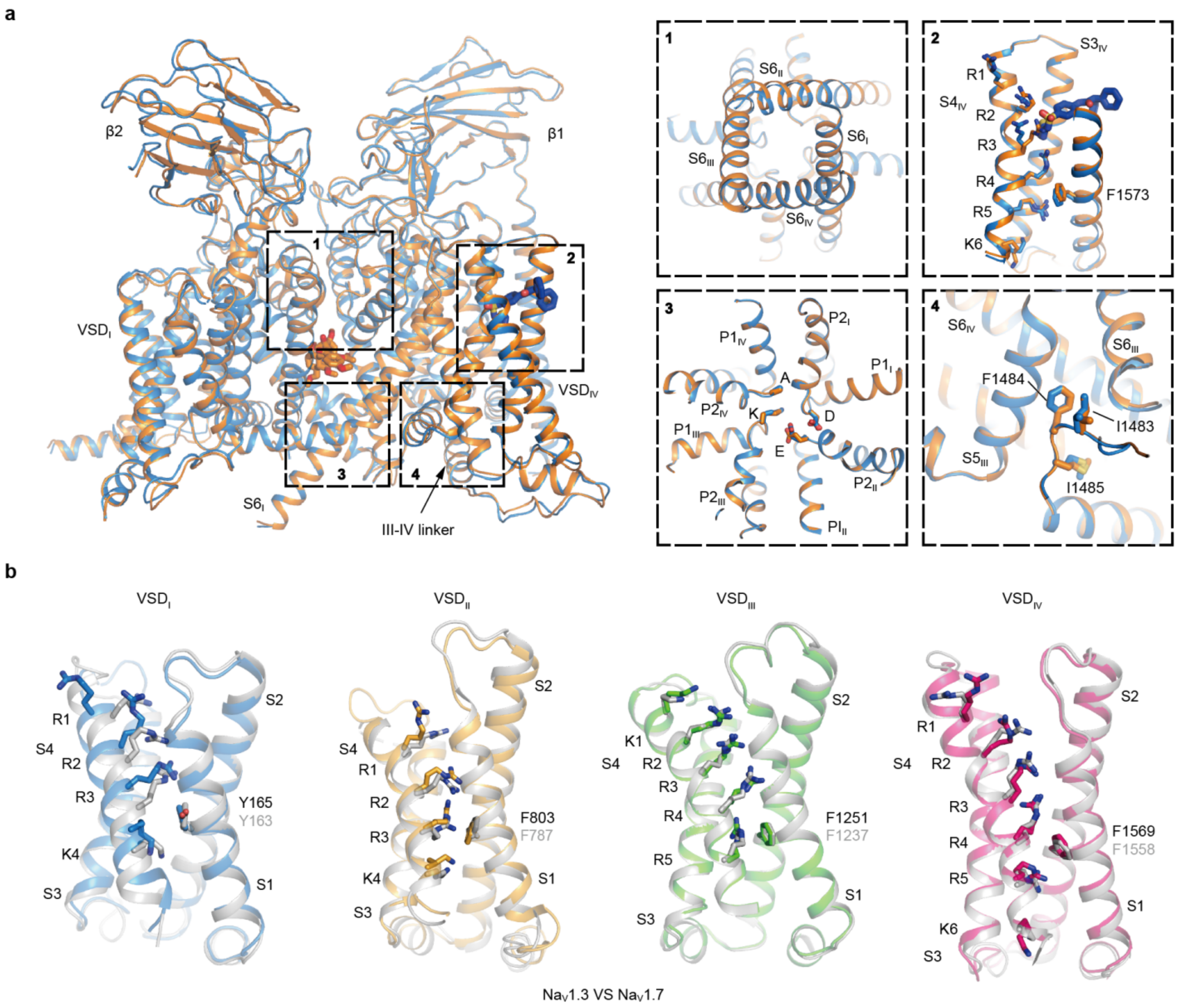

Supplementary Figure 6 Structural comparison of Na_V1.3.

a. Overall structural superposition of the Na_V1.3-BLA (orange) and Na_V1.3-ICA (blue). On the right, four panels showing the comparison of the activation gate, VSD_{IV}, selectivity filter and fast inactivation gate of the two structures, respectively. **b.** VSDs of Na_V1.3 are superimposed with that of Na_V1.7 (grey). Gating charges are shown in sticks.

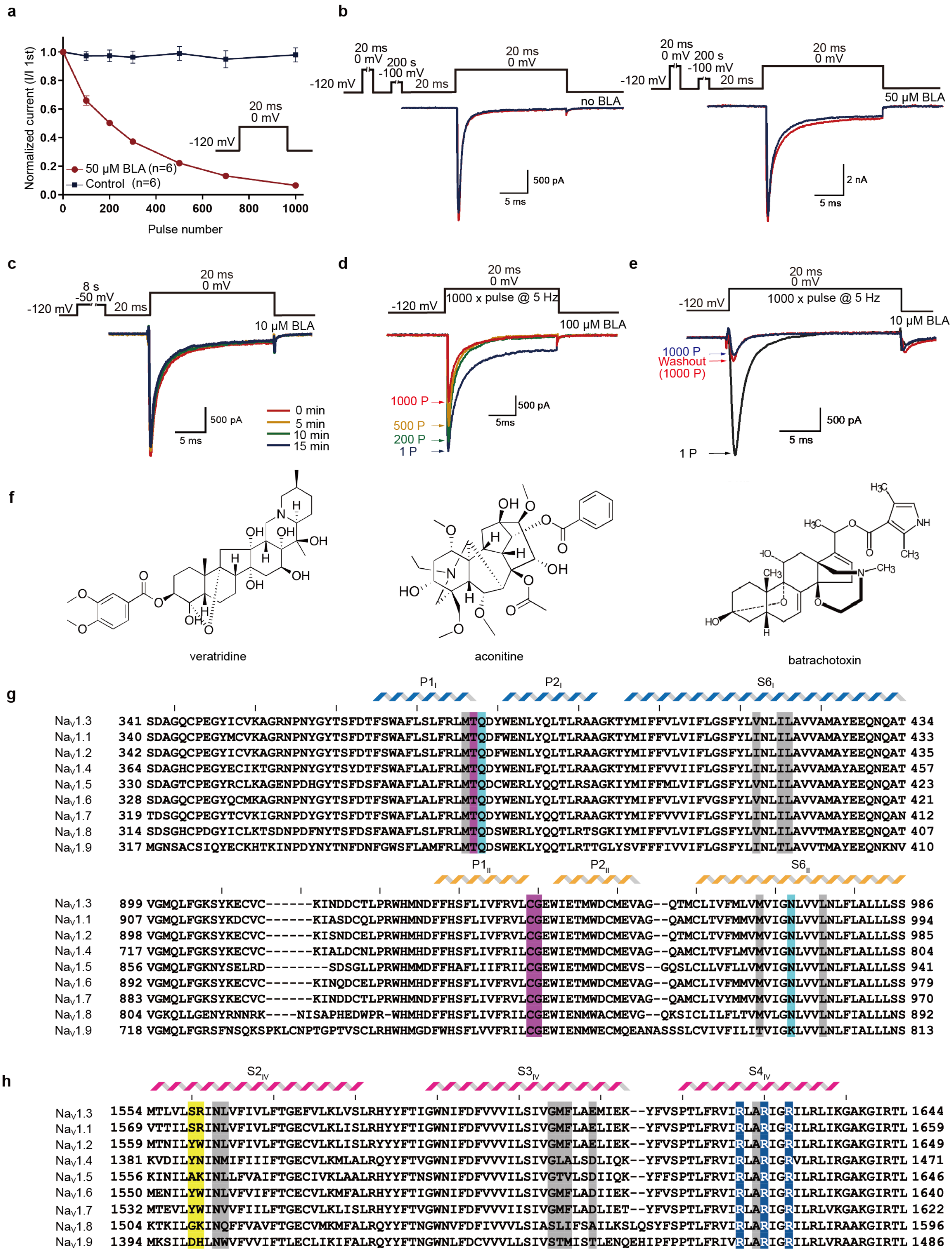

Supplementary Figure 7 Functional study of the NaV1.3 modulated by BLA and chemical structures of site-2 neurotoxins.

a. BLA inhibits NaV1.3 in use-dependent manner. Use-dependent block by BLA after 20-ms pulses at 5 Hz from −120 mV to 0 mV (no BLA, squares; 50 μM BLA, circles; data are mean ± SEM; n = 6). **b.** BLA has no effect on NaV1.3 in resting state. Representative traces of NaV1.3 were recorded from holding for -200 s at -

100 mV without or with 50 µM BLA in the bath solution, respectively. A 20 ms test pulse at 0 mV was first recorded as a control trace (blue trace) from holding potential of -120 mV, then the patched cells were holding for 200 s at -100 mV, after 20 ms back to -120 mV, a second 20 ms test pulse at 0 mV was recorded (red trace) for either without or with 50 µM BLA. Similar current traces were recorded from 5 cells with BLA and from 4 cells without BLA. **c.** BLA has no inhibitory effect on inactivated $Na_V1.3$. The inactivated-state inhibition protocol was composed of an 8-s pre-pulse condition step at -50 mV to inactivate the channels followed by a 20-ms test pulse at 0 mV from the holding potential of -120 mV. $Na_V1.3$-transfected HEK293T cells were measured using the protocol when the cells were incubated in bath solution containing 10 µM BLA for 0 min (black), 5 min (blue), 10 min (orange) and 15 min (purple). The number of cells measured for each waiting time were 6-10. **d.** Use-dependent inhibition of $Na_V1.3$ by BLA in intracellular solution. Representative current traces shown use-dependent inhibition of $Na_V1.3$ by 100 µM BLA in the intracellular solution. Current were elicited by a 20 ms test pulse at 0 mV from holding potential of -120 mV for 1000 repetitive pulses at 5 Hz. Similar current traces were recorded from 4 cells, yielding a mean inhibition of 43.6% ± 0.8%, n=4. **e.** BLA binding is nearly irreversible to $Na_V1.3$. After 1000 repetitive pulses to reach steady-state inhibition of $Na_V1.3$ by 10 µM BLA (1000 P, blue trace), the bath solution was exchanged with fresh bath solution without BLA, after waiting for 5-10 min, a second train of 1,000 repetitive pulses were recorded from the same patched cell. After the second 1,000 repetitive pulses (washout-1000P, red trace), the current was only recovered by less than 5% of the peak current (mean 4.8% ± 1.2%, n=6). The number of cells measured for the wash-out experiment were 6. **f.** Chemical structures of veratridine, aconitine and batrachotoxin. **g.** The sequence alignments of P1, P2 and S6 of domain I and domain II among the human NaV channels. Residues contributed to BLA binding are shaded. Especially, residues whose carbonyl oxygens and side chains forming hydrogen bonds with BLA are highlighted in magenta and cyan, respectively. **h.** The sequence alignments of S2, S3 and S4 of domain IV among the human NaV channels. The residues involved in forming receptor site of ICA are shaded. The key determinants S1559 and R1560 for ICA selectivity are highlighted in yellow. The three gating charges (R2-R4) directly interacting with ICA are highlighted in blue. Other conserved residues are shaded in grey. Source data are provided as a Source Data file.

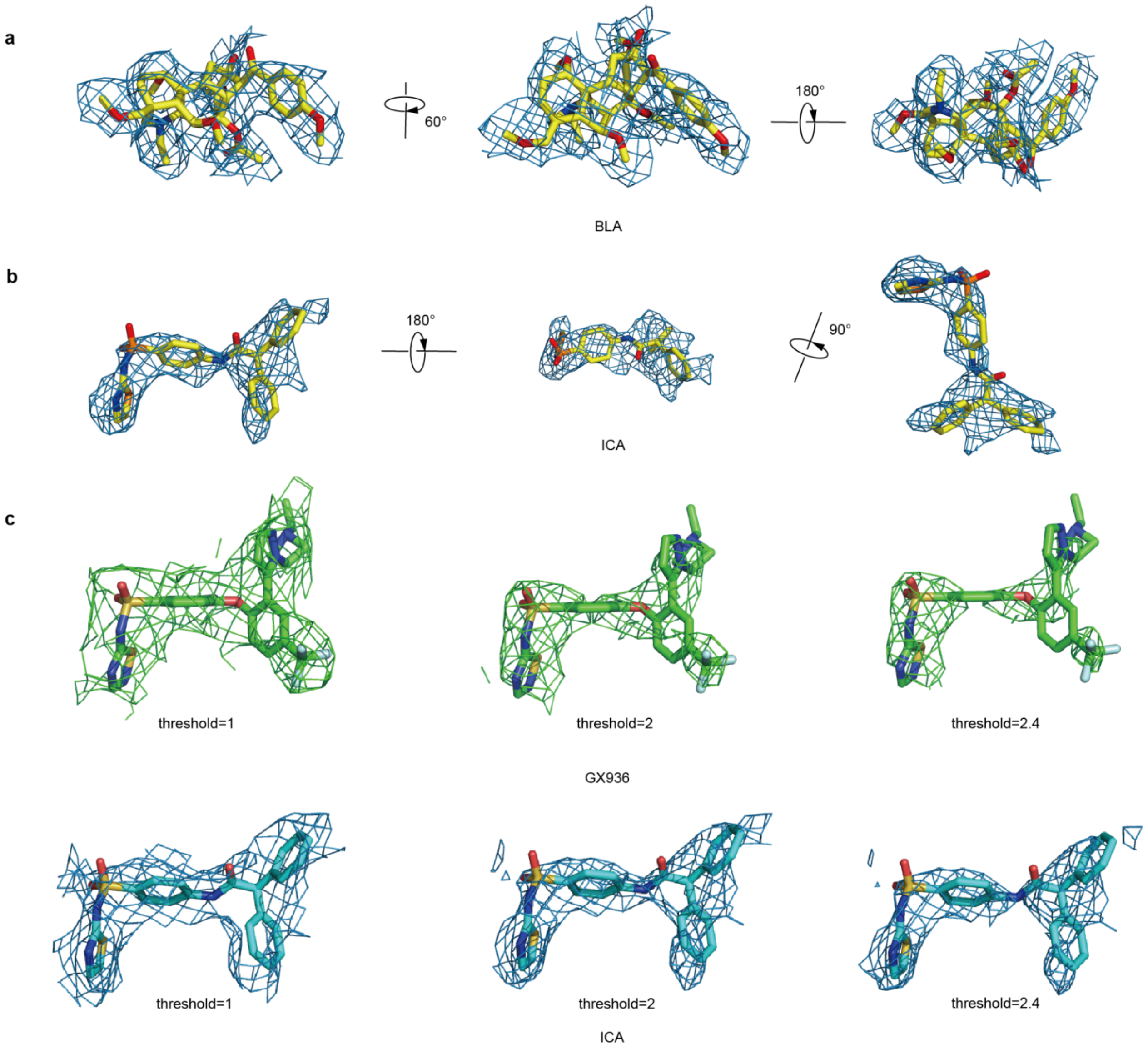

Supplementary Figure 8 Density maps for sodium channel antagonists.

a. The cryo-EM density maps for BLA presented from three different angles contoured at 3σ. **b.** The cryo-EM density maps for ICA presented from three different angles contoured at 2.4σ. **c.** The X-ray electron density maps of GX-936 (PDB code: 5EK0) and the cryo-EM density map for ICA contoured at 1σ, 2σ, and 2.4σ, respectively.

Supplementary Table 1. Structure and sequence identity among subtypes of Na_Vs

Na_V1.3/β1/β2 vs.	RMSD (Å）	Identity	No. of aligned residues
Na_V1.1	1.37	90.6%	1138
Na_V1.1/β2	1.67	88.0%	1249
Na_V1.2	1.46	92.6%	1101
Na_V1.2/β2	1.52	92.6%	1214
Na_V1.4	1.39	82.8%	1099
Na_V1.4/β1	1.47	84.8%	1267
Na_V1.5	1.49	77.7%	1073
Na_V1.7	1.37	83.6%	1092
Na_V1.7/β1	1.4	85.7%	1265
Na_V1.7/β1/β2	1.75	85.7%	1293

Supplementary Table 2. Primers used in this study.

Name	Sequence
Na_V1.3-F	ACAGCTCTTAAGGGATCCCGGTCCGATGGCACAGGCACTGTTGGT
Na_V1.3-R	GGAACAGAACTTCCAGTGCGGCCGCCTTTTGATTTTCTCTGACCTCTTTTCCTTTGC
β1-F	ACAGCTCTTAAGGGATCCCGGTCCGATGGGGAGGCTGCTGGCCTTA
β1-R	GGAACAGAACTTCCAGTGCGGCCGCTTCGGCCACCTGGACGCCCGTG
β2-F	ACAGCTCTTAAGGGATCCCGGTCCGATGCACAGAGATGCCTGGCTA
β2-F	TTGTCGAGACTGCAGGCTCTAGATCACTTGGCGCCATCATCCGGGTTGCCTTC

Supplementary Table 3. Cryo-EM data collection, refinement and validation statistics

	Na_V1.3/β1/β2-ICA (EMDB-32343) (PDB: 7W7F)	Na_V1.3/β1/β2-BLA (EMDB-32341) (PDB: 7W77)
**Data collection and processing**		
Magnification	105,000 ×	105,000 ×
Voltage (kV)	300	300
Electron exposure (e^{-}/Å^2)	60	60
Defocus range (μm)	−1.2 ~ −2.2	−1.2 ~ −2.2
Pixel size (Å)	1.04	1.04
Symmetry imposed	C1	C1
Initial particle images (no.)	1,088,844	1,403,849
Final particle images (no.)	175,513	152,431
Map resolution (Å)	3.35	3.30
FSC threshold	0.143	0.143
Map resolution range (Å)	3.0 ~ 5.0	3.0 ~ 5.0
**Refinement**		
Initial model used (PDB code)	6J8E,6J8H	Na_V1.3/β1/β2-ICA
Model resolution (Å)	3.70	3.48
FSC threshold	0.5	0.5
Map sharpening *B* factor (Å^2)	-90	-97
Model composition		
Non-hydrogen atoms	12,051	12,054
Protein residues	1,424	1,424
Ligands	31	30
B factors (Å^2)		
Protein	66.67	38.44
Ligand	58.56	37.81
R.m.s. deviations		
Bond lengths (Å)	0.006	0.004
Bond angles (°)	0.730	0.793
Validation		
MolProbity score	3.03	2.82
Clashscore	14	13
Poor rotamers (%)	0.00	0.30
Ramachandran plot		
Favored (%)	91.45	91.74
Allowed (%)	8.48	8.26
Disallowed (%)	0.07	0.00
