# Supplementary material for: Structural basis for modulation of human NaV1.3 by clinical drug and selective antagonist
Source: Nat Commun. 2022 Mar 11;13:1286. doi: 10.1038/s41467-022-28808-5 (PMC8917200; doi:10.1038/s41467-022-28808-5)
Supplement: Supplementary file 3 — Reporting Summary [file 41467_2022_28808_MOESM3_ESM.pdf]

nature portfolio

Corresponding author(s): Daohua Jiang & Yan Zhao

Last updated by author(s): Jan 23, 2022

Reporting Summary

Nature Portfolio wishes to improve the reproducibility of the work that we publish. This form provides structure for consistency and transparency in reporting. For further information on Nature Portfolio policies, see our Editorial Policies and the Editorial Policy Checklist.

Statistics

For all statistical analyses, confirm that the following items are present in the figure legend, table legend, main text, or Methods section.

n/a	Confirmed	
☐	☒	The exact sample size (*n*) for each experimental group/condition, given as a discrete number and unit of measurement
☐	☒	A statement on whether measurements were taken from distinct samples or whether the same sample was measured repeatedly
☒	☐	The statistical test(s) used AND whether they are one- or two-sided<br>*Only common tests should be described solely by name; describe more complex techniques in the Methods section.*
☒	☐	A description of all covariates tested
☐	☒	A description of any assumptions or corrections, such as tests of normality and adjustment for multiple comparisons
☐	☒	A full description of the statistical parameters including central tendency (e.g. means) or other basic estimates (e.g. regression coefficient) AND variation (e.g. standard deviation) or associated estimates of uncertainty (e.g. confidence intervals)
☒	☐	For null hypothesis testing, the test statistic (e.g. *F*, *t*, *r*) with confidence intervals, effect sizes, degrees of freedom and *P* value noted<br>*Give P values as exact values whenever suitable.*
☒	☐	For Bayesian analysis, information on the choice of priors and Markov chain Monte Carlo settings
☒	☐	For hierarchical and complex designs, identification of the appropriate level for tests and full reporting of outcomes
☒	☐	Estimates of effect sizes (e.g. Cohen's *d*, Pearson's *r*), indicating how they were calculated

Our web collection on statistics for biologists contains articles on many of the points above.

Software and code

Policy information about availability of computer code

Data collection	All cryo-EM data were collected using SerialEM 3.8 , electrophysiology data were collected using PatchMaster v2x90.5.
Data analysis	Collected cryo-EM data were processed using RELION3.0, MotionCor2, Gctf, cryoSPARC v3.2.0, cisTEM 1.0.0-beta. Map and model refinement were processed with UCSF-Chimera v1.14, COOT v0.8.9.2, Phenix v1.17.1. Electrophysiology data were processed using Igor 6.2. All figures were made using PyMOL 2.5.1 and Prism 8.0.1.

For manuscripts utilizing custom algorithms or software that are central to the research but not yet described in published literature, software must be made available to editors and reviewers. We strongly encourage code deposition in a community repository (e.g. GitHub). See the Nature Portfolio guidelines for submitting code & software for further information.

Data

Policy information about availability of data

All manuscripts must include a data availability statement. This statement should provide the following information, where applicable:

- Accession codes, unique identifiers, or web links for publicly available datasets
- A description of any restrictions on data availability
- For clinical datasets or third party data, please ensure that the statement adheres to our policy

The sequences of human Nav1.3, β1 and β2 are available in the following links:
Nav1.3(UniProt ID: Q9NY46-2): https://www.uniprot.org/uniprot/Q9NY46-2
β1 (UniProt ID: Q07699): https://www.uniprot.org/uniprot/Q07699
β2 (UniProt ID: O60939): https://www.uniprot.org/uniprot/O60939
The novel structures determined in this study are deposited in PDB Bank and Electron Microscopy Data Bank (EMDB):

Nav1.3-β1-β2-BLA: 7W77; EMD-32341
Nav1.3-β1-β2-ICA: 7W7F; EMD-32343

Field-specific reporting

Please select the one below that is the best fit for your research. If you are not sure, read the appropriate sections before making your selection.

☒ Life sciences ☐ Behavioural & social sciences ☐ Ecological, evolutionary & environmental sciences

For a reference copy of the document with all sections, see nature.com/documents/nr-reporting-summary-flat.pdf

Life sciences study design

All studies must disclose on these points even when the disclosure is negative.

Sample size	Sample sizes were not predetermined for this study. For the cryo-EM studies, the number of micrographs is determined by the available microscope time. For the electrophysiology experiments, the authors have recorded 4 or more cells/patches, which are sufficient enough to see real effects of the mutations based on the reproducibility of the results. The sample sizes of all these experiments were determined based on the consistency and variability.
Data exclusions	For cryo-EM analysis, micrographs with CTF fitting worse than 5 angstrom were discarded. Following cryo-EM data processing only kept high-resolution and homogeneous particles to generate the final high resolution map.
Replication	Sample preparation related experiments including purification and SDS-PAGE gel electrophoresis were reproduced at least three times independently. Whole cell patch clamp recording were reproduced with about 6 different cells. All attempts at replication were successful.
Randomization	All particles were aligned to a resolution low-passed initial model, and divided randomly into several classes. As the translation and rotation operations went precisely, homogeneous particles went to the same class. For electrophysiology experiments, randomization is not relevant as no group allocations were performed.
Blinding	The investigators were not blinded as the parameters for cryo-EM analysis and electrophysiology experiments did not require subjective assessments of the treatments or their outcomes that might otherwise influence the validity of the results.

Reporting for specific materials, systems and methods

We require information from authors about some types of materials, experimental systems and methods used in many studies. Here, indicate whether each material, system or method listed is relevant to your study. If you are not sure if a list item applies to your research, read the appropriate section before selecting a response.

Materials & experimental systems

n/a	Involved in the study
☒	☐ Antibodies
☐	☒ Eukaryotic cell lines
☒	☐ Palaeontology and archaeology
☒	☐ Animals and other organisms
☒	☐ Human research participants
☒	☐ Clinical data
☒	☐ Dual use research of concern

Methods

n/a	Involved in the study
☒	☐ ChIP-seq
☒	☐ Flow cytometry
☒	☐ MRI-based neuroimaging

Eukaryotic cell lines

Policy information about cell lines

Cell line source(s)	FreeStyle 293-F cells (Gibco, USA); 293-T cells (Gibco, USA); Sf9 (Gibco, USA)
Authentication	We did not anthenticate them.
Mycoplasma contamination	The cell lines were not tested for mycoplasma contamination.
Commonly misidentified lines (See ICLAC register)	No other cell line used in this study.
