# Supplementary figures and images for: Structural basis for modulation of human NaV1.3 by clinical drug and selective antagonist
Source: Nat Commun. 2022 Mar 11;13:1286. doi: 10.1038/s41467-022-28808-5 (PMC8917200; doi:10.1038/s41467-022-28808-5)

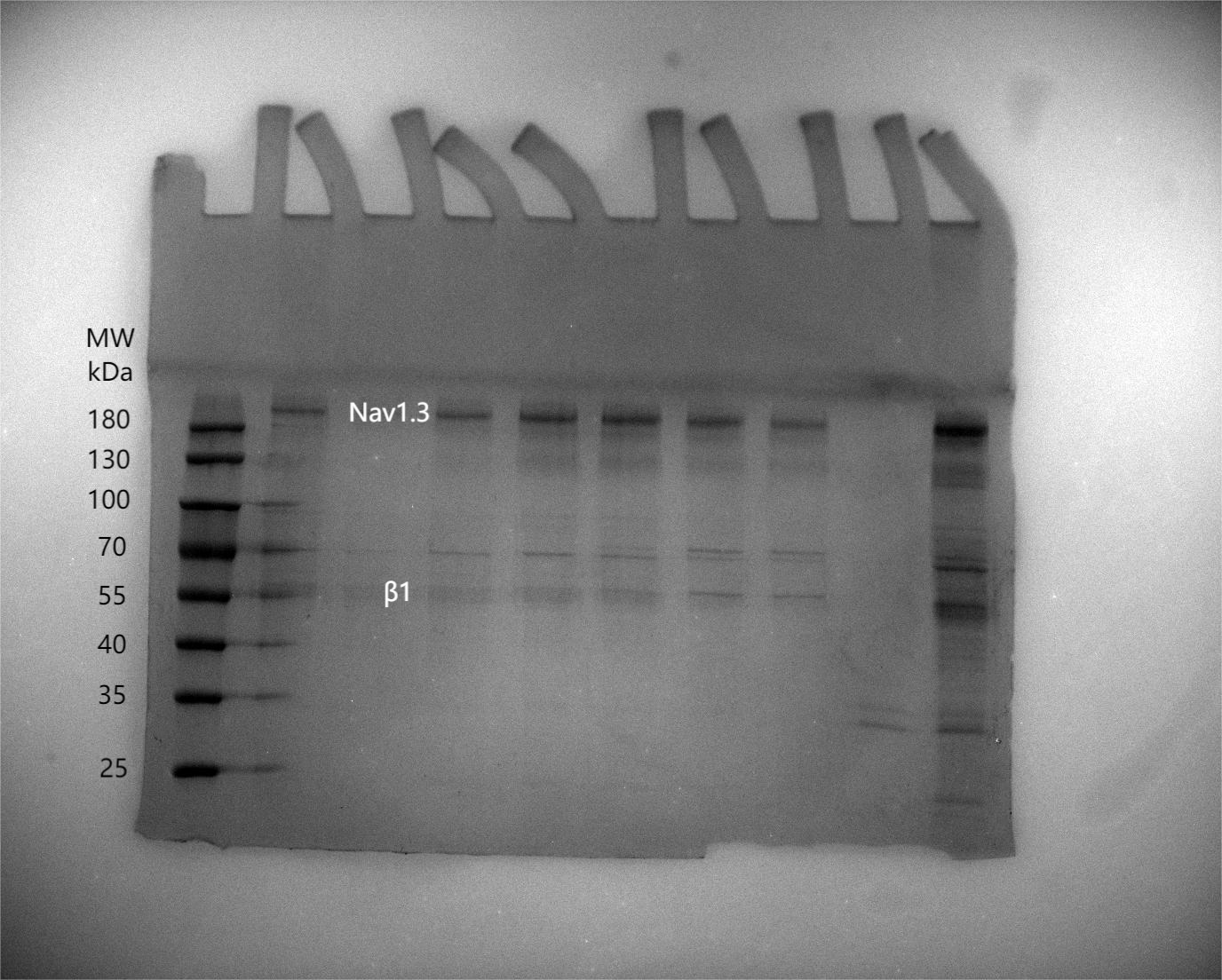

Supplement: Supplementary file 4 — Source Data [file 41467_2022_28808_MOESM4_ESM.zip › SourceData_Supplementary Fig1b.png]
